# Horsehair as a Substitute for Wire

**Published:** 1866-04

**Authors:** Thomas Smith

**Affiliations:** Demonstrator of Anatomy at St. Bartholomew’s Hospital, and Assistant Surgeon to the Hospital for Sick Children


5. *Horsehair as a Substitute for Wire.*—By THOMAS SMITH, Esq., Demonstrator of Anatomy at St. Bartholomew's Hospital, and Assistant Surgeon to the Hospital for Sick Children.

Experience has ere this fully justified the claims put forward by Dr. Marion Sims, Dr. Simpson, and others, for the advantages of the metalic suture over threads of organic origin. For ordinary purposes, the cleanliness, security, and unirritating nature of silver wire leave but little to be desired in the way of improvement in material, and though in most cases such sutures answer all purposes for which they are designed, yet to some localities and to certain tissues they are not well suited. The adjustment of wire sutures to wounds of the eye-lid, foreskin, scrotum, and other parts where the integuments are lax and delicate in texture, is difficult, and their withdrawal is painful, and liable to cause rending and laceration of the freshly united edges. The difficulty of introduction consists in the laxity of the parts, disposing the edges of the wound to twist and coil up far more readily than ends of the wire; while the withdrawal of the sutures is painful, owing to the rigid condition the wire assumes before its removal, and this occurs notwithstanding every precaution that may be taken to anneal it previous to its introduction. In allusion to this inconvenience, Dr. Mott, in his work "On Surgery," in speaking of metallic sutures, makes the remark: "The introduction of wire sutures is easy enough; the withdrawal is often attended with great inconvenience, and even with risk of tearing the imperfectly united edges of the wound asunder." (Vol. i., p. 335.)

With a view of finding a material for sutures as unirritating and as unabsorbent as wire, but more easy of adjustment and withdrawal, I performed during last spring a series of experiments on animals, to determine the suitability of horsehair as a substitute for wire in certain cases. The horsehair used was such as is ordinarily sold, by fishing-tackle makers. The experiments were performed upon dogs. The general results showed that there was no appreciable difference shown by the tissues in their tolerance of silver wire and horsehair. Both materials were equally unirritant; yet there was a difference in favor of the horsehair in the great facility of its adjustment and subsequent removal.

For the comparison between silk and horsehair, as illustrating the relative merits of the two materials for sutures, I venture to refer to the following experiments:

June 10th, 1861. Two wounds of equal length, dividing the entire thickness of the integuments, were made on opposite and corresponding parts of a dog's abdomen; four sutures were applied at equal intervals to each, horsehair being used to one wound, and fine ligature silk to the opposite. On the third day, both wounds looked alike healthy, and having their edges in close contact. On the fifth day, the edges of the wound with silk sutures were slightly reddened, and pouting a little between the points of suture; the opposite wound had united without suppuration. On the eighth day, three out of the four silk stitches had cut their way out, and the next day the remaining one came away, leaving the edges of the wound just separated, but granulating healthily. Three days later the wound had almost entirely healed. At this time the opposite sutures, which remained *in situ*, exciting no irritation whatever, until the dog's death, a month after the commencement of the experiment.

May 3rd. The femoral arteries of a dog were exposed to the same extent, just below Poupart's ligament. Around the vessel on the right lower limb was passed a stout horsehair, and loosely tied, a suture being similarly adjusted around the opposite artery. A month after the operation the wound on the right side was all but healed, and was secreting a little serous discharge. At the same time the wound on the left side was swollen, its edges were everted and inflamed, and had a profuse sanio-purulent discharge. Two days later the wound on the right side had healed around the track of the horsehair seton, which was retained, while around the silk on the other side there was profuse suppuration; the surrounding parts were red, tender, and much swollen; and as the animal's general health was suffering, and was rapidly emaciating, the silk was withdrawn. The wound now speedily altered its character, and by June 20th was soundly healed. September 3rd, four months after its introduction, the horsehair still remained around the right femoral artery, exciting no irritation, the parts being soundly healed around the track of the seton.

The unirritating nature of horsehair, as a material for suture, is no less marked when applied to the tissues of the human body. It was used by Mr. Paget in a case of double entropion, the wound of the operation being in one eyelid secured with horsehair sutures, while the opposite was brought together with fine sewing cotton. At the end of the week, three out of the four cotton sutures had cut out, while at the same time all four horsehair sutures remained firm.

As a material for attaching the margins of the skin, and mucous membrane after circumcision, or other operations for phimosis, I have found horsehair most useful, having employed it both in children and adults. In one case particularly, where a complete circumcision of the foreskin, with a free division of the mucous membrane, was performed on a middle-aged gentleman, its good effect was remarkable. Six sutures were introduced, and excited so little disturbance that the patient was not kept for a single day from his business, which involved pretty active exercise. The wound healed without suppuration, and though left in, at the patient's request, some of them for fourteen days, the sutures caused no irritation, and were removed at last without difficulty. In the removal, the advantage of horsehair sutures over the wire is considerable, since, unlike wire, which, after remaining a few days in a wound, stiffens into a metallic ring, horsehair, when cut just aside the knot, either retaining its original elasticity, springs open, or if it has been long soaked in the wound secretions, it becomes soft and pliable. I would recommend this suture for wounds of the eyelid and other parts of the face, and for the loose integuments of the scrotum and penis; since to all these parts I have either applied the suture myself with good effect, or I have seen it used by others at my suggestion.

But I can imagine that there are other uses to which it might be extended, and especially to facilitate the union of wounds of the conjunctiva. For the purpose of suture, long white tail hairs are the best. Before being used they should be soaked for a minute or two in water, or they may be drawn once or twice through the moistened finger-ends. The suture may be fastened with a double knot, but if the hair is stiff, a third knot is often required. It may be removed in the ordinary manner, seizing the knot with the forceps, and dividing the suture just aside of it. It is scarcely necessary to remark, that horsehair, as a suture, is not suitable for wounds, where there are much tension between the edges.---*Lancet.*

